# HHLA2 and PD-L1 co-expression predicts poor prognosis in patients with clear cell renal cell carcinoma

**DOI:** 10.1136/jitc-2019-000157

**Published:** 2020-01-19

**Authors:** Qiang-Hua Zhou, Kai-Wen Li, Xu Chen, Hai-Xia He, Sheng-Meng Peng, Shi-Rong Peng, Qiong Wang, Ze-An Li, Yi-Ran Tao, Wen-Li Cai, Ran-Yi Liu, Hai Huang

**Affiliations:** 1 Department of Urology, Sun Yat-Sen Memorial Hospital, Sun Yat-sen University, Guangzhou, Guangdong, China; 2 Guangdong Provincial Key Laboratory of Malignant Tumor Epigenetics and Gene Regulation, Sun Yat-Sen Memorial Hospital, Sun Yat-Sen University, Guangzhou, Guangdong, China; 3 State Key Laboratory of Oncology in South China & Collaborative Innovation Center of Cancer Medicine, Sun Yat-sen University Cancer Center, Guangzhou, Guangdong, China; 4 Department of Medical Oncology, Sun Yat-sen University Cancer Center, Guangzhou, China; 5 Department of Radiology, Massachusetts General Hospital, Harvard Medical School, Boston, Massachusetts, USA

**Keywords:** immunology, urology

## Abstract

**Background:**

Although clear cell renal cell carcinoma (ccRCC) is well known as a highly immunogenic tumor, only a small subset of patients could benefit from current immunotherapy, which might be due to the heterogeneity of immune microenvironment in ccRCC. So, it is meaningful to explore novel immunotherapy or combination therapy for improving therapeutic efficacy. HHLA2, a newly discovered B7 family member, is prevalently expressed in numerous tumors, including ccRCC. This study aimed to investigate the prognostic impact of HHLA2/PD-L1 co-expression and its relationship with tumor-infiltrating lymphocytes (TILs).

**Methods:**

The expression levels of HHLA2, PD-L1, CD8, and CD4 in cancer tissues from cases (206 in the training cohort and 197 in the validation cohort) with surgically resectable primary ccRCC were evaluated by immunohistochemistry.

**Results:**

The positive rates of HHLA2 were much higher than those of PD-L1 in ccRCC tissues. HHLA2-positive expression was significantly associated with necrosis, microvascular invasion, advanced Fuhrman nuclear, and TNM stage and indicated a shorter progression-free survival (PFS) and overall survival (OS) in both cohorts. Moreover, patients with HHLA2/PD-L1 co-expression suffered the highest risk of disease progression and death by a significant margin. Besides, HHLA2/PD-L1 co-expression was significantly associated with a high density of CD8^+^ and CD4^+^ TILs. Notably, a new immune classification, based on HHLA2/PD-L1 co-expression and TILs, successfully stratified PFS and OS, especially in patients with TILs positivity.

**Conclusions:**

The expression of HHLA2 is more frequent than PD-L1 in ccRCC. HHLA2/PD-L1 co-expression had an adverse impact on the prognoses of patients with ccRCC; this finding provides a rationale for combination immunotherapy with anti-HHLA2 and PD-L1 blockage for patients with ccRCC in the future.

## Background

Clear cell renal cell carcinoma (ccRCC) represents the most common renal cell cancer (RCC), which accounts for 2% of the global cancer burden.^1–3^ In 2013 alone, >140 000 patients, especially advanced patients, died due to either renal carcinoma or corresponding complications despite the development of multidisciplinary treatments for ccRCC (including radical or partial nephrectomy and targeted therapy).^4^ Therefore, there is a critical clinical need to develop innovative strategies to reverse poor clinical outcomes associated with current therapies in ccRCC.

Since the last century, Immunotherapy, such as anti-CTLA-4 antibody^5^ and anti-PD-1 antibody,^6^ has been regarded as a promising therapy for ccRCC due to its immunogenic nature. In particular, anti-programmed death 1 (PD-1) antibody nivolumab was approved by the Food and Drug Administration (FDA) because of a higher response rate and less frequent adverse events compared with mTOR inhibitor everolimus in a phase III trial.^6^ However, the objective response rates were only 25%–42% with nivolumab or even combined with ipilimumab (anti-CTLA-4) in advanced RCC, indicating alternative immunosuppressive checkpoints or pathways disrupting immunosurveillance in ccRCC.^5 6^ In this perspective, it is of great significance for the guidance of clinical immunotherapy to establish a suitable immunophenotyping system to predict patients who will respond to immune checkpoint blockades^7^ .

According to the theory proposed by Teng *et al*, a tumor is classified into four groups based on the TIL density and programmed death 1 ligand 1 (PD-L1) expression, including adaptive immune resistance, immunological ignorance, tolerance, and intrinsic induction.^8^ Although this classification could provide guidance for immunotherapy, some misadvises would also arise as only PD-L1 but no other immunosuppressive checkpoints were included in the theory. Besides PD-L1, other negative regulatory co-stimulatory molecules including PD-L2 and B7-H3 were detected with high expression and associated with poor prognosis in ccRCC, whereas the positive rates of PD-L2 and B7-H3 were limited.^9 10^ Furthermore, the expression of a PD-L1 and/or PD-L2 on tumor cells, as well as a low percentage of CD8^+^PD-1^+^ T cells might be valuable for predicting response to PD-1 blockade therapy in metastatic ccRCC.^11^ Simultaneous activation of CD8^+^ T cells and PD-1 blockade provides more benefits for metastatic ccRCC.^12^ However, tumor-infiltrating naïve CD4 T cells are associated with low responsiveness to PD-1 blockades.^13^ Collectively, the theory, mentioned previously, could not fully clarify the complexity of the immune microenvironment in ccRCC. Therefore, it will be of important clinical significance to optimize existing immune classifications by introducing new immunosuppressive checkpoints with a high positive rate in ccRCC.

Recently, increasing focus has turned to human endogenous retrovirus-H long terminal repeat-associating protein 2 (HHLA2, also known as B7-H7), a newly discovered member of the B7 family and analogous to PD-L1, PD-L2, and B7-H3.^14^ HHLA2 inhibits the proliferation and cytokine production of CD4^+^ and CD8^+^ T cells through binding to its putative receptors in a variety of immune cells.^14 15^ To date, transmembrane and immunoglobulin domain containing protein 2 (TMIGD2) is the only evidenced receptor of HHLA2.^16^ As TMIGD2 is mainly expressed on naive T cells but not mature T cells, it would be possible that other undefined molecules are responsible for the immunosuppressive role of HHLA2.^17^ In addition, Rahimi *et al* reported that TMIGD2 was also detected in endothelial cells, therefore, HHLA2 may also have a potential role in tumor angiogenesis.^18^ Janakiram *et al* demonstrated that HHLA2 was widely expressed in cancer samples such as breast, lung, and prostate cancers.^16^ Moreover, HHLA2 was more prevalently expressed in various cancer cells than PD-L1 and HHLA2 overexpression was common in PD-L1-negative breast cancer and cholangiocarcinoma.^19 20^ HHLA2 was also reported to be overexpressed in RCC, compared with normal renal tissue, and the expression of HHLA2 was associated with poor prognosis of RCC.^21 22^ However, the relationship between HHLA2 and the immune microenvironment has not been uncovered in RCC.

In our present study, we evaluated the relationship between HHLA2 expression, clinicopathological features, and the immune microenvironment by analyzing date from two large cohorts. Then, we introduced HHLA2 expression status into the immune classification based on TIL density and PD-L1 expression to optimize the present immune classification and establish a novel immunophenotyping system. We then examined its clinical significance for ccRCC in two independent cohorts. This study may provide a useful guide for patients with ccRCC in choosing proper immunotherapy.

## Materials and methods

### Patients and samples

On approval by the Institutional Ethical Boards of Sun Yat-sen University Cancer Center (SYSUCC) and Sun Yat-sen Memorial Hospital (SYMH), we retrospectively analyzed data from two cohorts: a training cohort from SYSUCC (206 patients) and a validation cohort from SYMH (197 patients). Patients in both cohorts underwent surgical resection for ccRCC from January 2006 to December 2013, and each patient signed informed consents. Patients who received neoadjuvant therapy were excluded from the present study. Formalin-fixed, paraffin-embedded (FFPE) blocks of all patients were collected from the pathology department and two senior pathologists were assigned to confirm Fuhrman nuclear grade, T stage and N status with H&E tumor slides, according to the American Joint Committee on Cancer (AJCC) 2009 TNM classification for ccRCC. Distant metastasis was evaluated by imaging examination. Progression-free survival (PFS) was defined as time span from the date of surgery to the date of cancer progression or death, and the overall survival (OS) was defined as time span from the date of surgery to the date of death. The follow-up was censored on 31 December 2018, the date of the last follow-up for patients without progression or death event.

### Immunohistochemistry

Immunohistochemistry (IHC) staining for HHLA2, PD-L1, CD8, and CD4 was accomplished by a professional pathologist.^23–25^ After deparaffinization, rehydration, antigen retrieval, endogenous peroxidase inactivation, and blocking non-specific binding, the 4 µM-thick sections were incubated with primary antibodies (anti-HHLA2: Sigma-Aldrich, HPA055478; anti-PD-L1: cell signaling technology, CST #13684; anti-CD8: CST, #85336; anti-CD4: Abcam, ab252199) at 4°C overnight. Then, the slides were incubated with a corresponding secondary antibody and visualized by using a DAKO EnVision Detection System (Dako). Finally, the slides were counterstained with hematoxylin, dehydrated, and cover-slipped.

### Quantification of HHLA2, PD-L1 and infiltration of T cells

HHLA2 and PD-L1 expressions on the tumor cell surface were evaluated based on the percentage of positive cells (eg, number of positive cells/ numbers of total cells). The optimal values for HHLA2 and PD-L1 expression were 20% and 10%, respectively, which was calculated with X-tlie. For CD8 and CD4 evaluation, the number of CD8^+^ or CD4^+^ TILs was counted and averaged over five high-power fields for each case.^26^

### Statistical analysis

The correlation analyses were performed using the χ^2^ test, whereas survival analysis was performed by the Kaplan-Meier method to depict the survival curves of PFS and OS. A log-rank test was carried out to examine intergroup differences. Univariate and multivariate analyses were executed via Cox proportional hazard model. All statistical analyses were accomplished by SPSS V.24.0 software and Graphpad Prism 7 software (La Jolla, California, USA). A p value <0.05 was considered statistically significant.

## Results

### Patient characteristics

The baseline patient clinicopathological characteristics of the two independent ccRCC cohorts are listed in the online supplementary table S1. In the training cohort, the median age at surgery was 53 years (ranging from 19 to 84 years). At the end of follow-up, 26.7% (55/206) patients had disease progression and 24.8% (51/206) patients died from ccRCC. The median PFS time was 47.7 months (ranging from 1 to 154 months) and the median OS time was 50.9 months (ranging from 1 to 154 months). In the validation cohort, the median age at surgery was also 53 years, (ranging from 21 to 80). At the end of follow-up, 30.5% (60/197) patients had disease progression and 27.4% (54/197) patients died from ccRCC. The median PFS was 57.6 months (ranging from 1 to 139 months) and the median OS was 61.3 months (ranging from 1 to 139 months).

10.1136/jitc-2019-000157.supp1 Supplementary data

**Figure 1:**
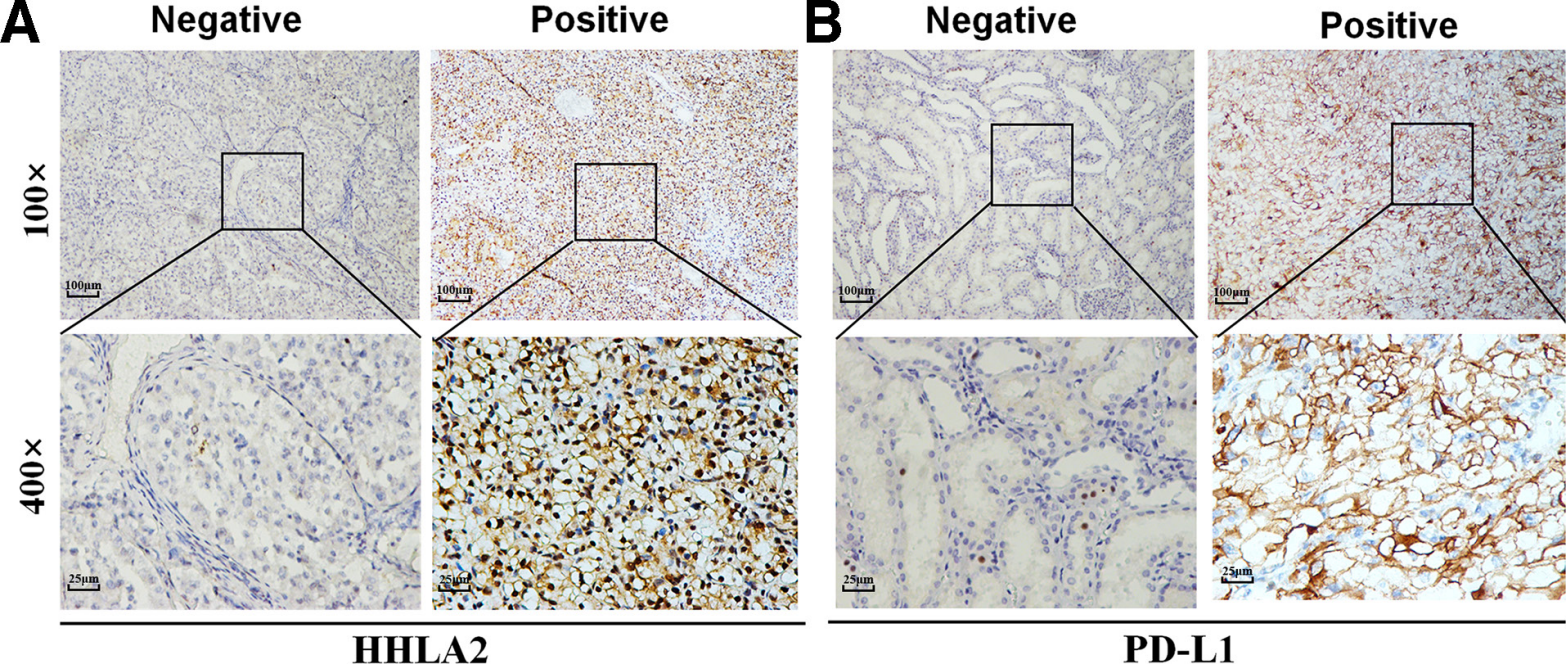
HHLA2 and PD-L1 expression in ccRCC tissue samples. Representative micrographs of HHLA2 (A) and PD-L1 (B) expression within the tumor. ccRCC, clear cell renal cell carcinoma; HHLA2, human endogenous retrovirus-H long terminal repeat-associating protein 2; PD-L1, programmed death 1 ligand 1.

Representative IHC photomicrographs for HHLA2 and PD-L1 in ccRCC are shown in figure 1. In both cohorts, the positive rates of HHLA2 expression were 44.2% (91/206) and 41.1% (81/197), which, interestingly enough, were significantly higher than the rates of PD-L1 expression at 33.0% (68/206) and 32.0% (63/197) (online supplementary table S1).

### Association of HHLA2 and PD-L1 expression with clinicopathological features

The association between HHLA2 expression and clinicopathological characteristics is illustrated in table 1. The HHLA2 positivity was significantly correlated with necrosis (p*=*0.001 for both cohorts), high Fuhrman grade (training cohort: p<0.001; validation cohort: p=0.032), and advanced TNM stage (training cohort: p<0.001; validation cohort: p=0.008) in both cohorts. Additionally, the HHLA2 positivity was associated with microvascular invasion (MVI) (p=0.035) in the training cohort, but not in the validation cohort. This significant association was also detected between HHLA2 and PD-L1 expression in both cohorts (training cohort: p=0.003; validation cohort: p=0.002).

**Table 1:** The association of HHLA2 expression level with clinicopathological characteristics in the training cohort and validation cohort

Variables	Training cohort (n=206)				Validation cohort (n=197)			
	Patients, n	HHLA2 expression			Patients, n	HHLA2 expression		
		Negative	Positive	P value		Negative	Positive	P value
Age (y)				0.619				0.254
<53	90	52	38		102	64	38	
≥53	116	63	53		95	52	43	
Gender				0.409				0.11
Male	134	72	62		141	88	53	
Female	72	43	29		56	28	28	
Necrosis				**0.001**				**0.001**
Absent	149	94	55		151	99	52	
Present	57	21	36		46	17	29	
MVI				**0.035**				0.148
Absent	179	105	74		169	103	66	
Present	27	10	17		28	13	15	
Fuhrman grade				**<** **0.001**				**0.032**
Low^1 2^	136	93	43		129	83	46	
High^3 4^	70	22	48		68	33	35	
TNM stage				**<** **0.001**				**0.008**
Low (I, II)	146	94	52		146	94	52	
High (III, IV)	60	21	39		51	22	29	
PD-L1				**0.003**				**0.002**
Negative	138	87	51		134	89	45	
Positive	68	28	40		63	27	36	

*P values <0.05 in bold are statistically significant.

HHLA2, human endogenous retrovirus-H long terminal repeat-associating protein 2; MVI, microvascular invasion; PD-L1, programmed death 1 ligand 1.

Similarly, in two cohorts, PD-L1 positivity was significantly associated with necrosis (p=0.041 and 0.001, respectively), high Fuhrman grade (p=0.002 and 0.003, respectively) and advanced TNM stage (p=0.003 and 0.002, respectively). A significant association between PD-L1 positivity and MVI was only identified in the training cohort (p<0.001) (online supplementary table S2).

### Impact of HHLA2 and PD-L1 expression on the progression-free survival and overall survival

**Figure 2:**
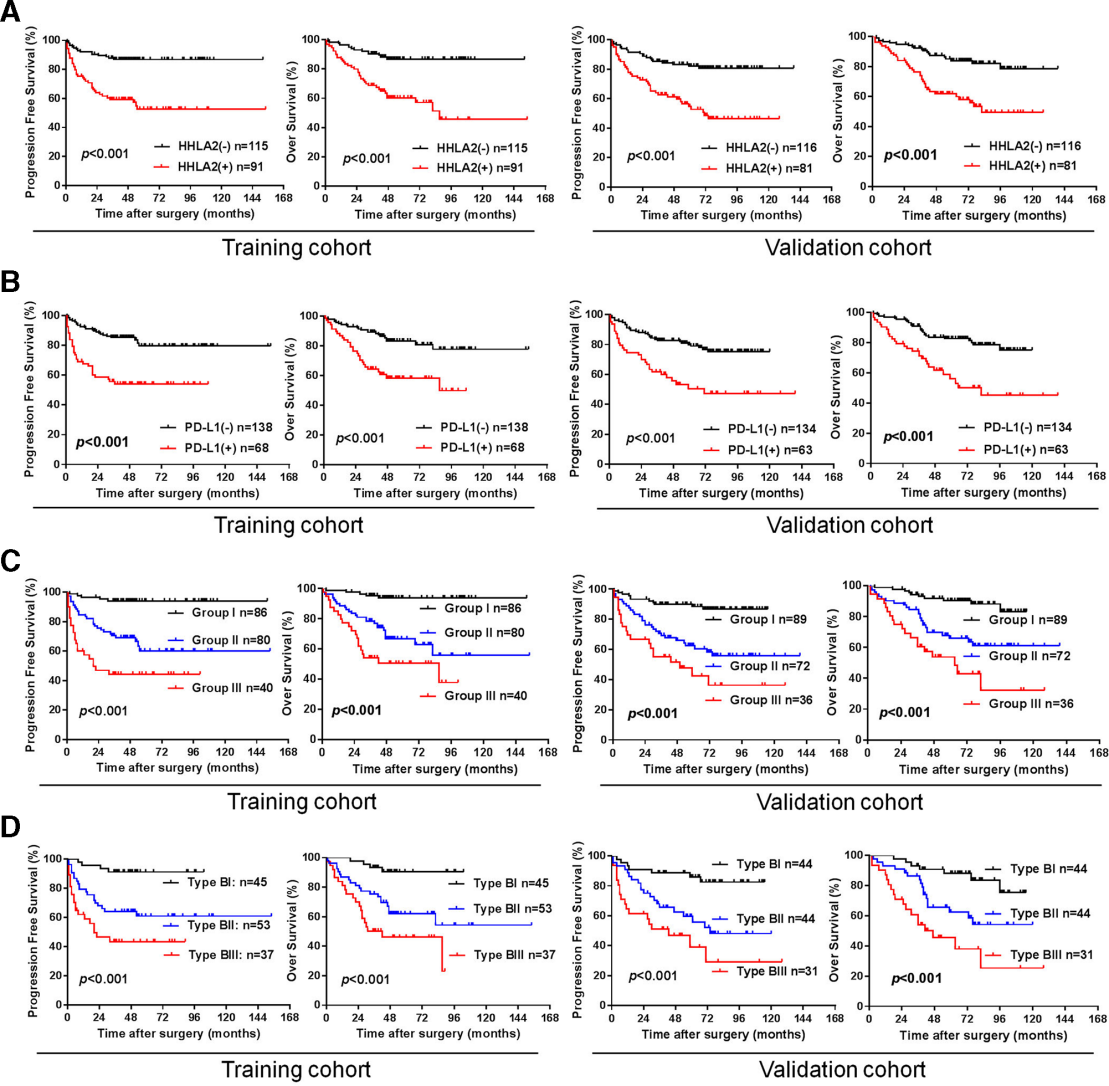
Kaplan-Meier survival curves for PFS and OS of patients with ccRCC according to HHLA2 and PD-L1 expression. (A) PFS and OS according to HHLA2 expression status in the training cohort and validation cohort. (B) PFS and OS according to PD-L1 expression status in the training cohort and validation cohort. (C) PFS and OS according to a combination of HHLA2/PD-L1 co-expression. Group I: HHLA2 (−)/PD-L1 (−); group II: HHLA2 (+)/PD-L1 (−) or HHLA2 (−)/PD-L1 (+); group III: HHLA2 (+)/PD-L1 (+). (D) PFS and OS according to new immune classification. Type BI: TILs positive and both negative (HHLA2 (−)/PD-L1 (−)); type BII: TILs positive and single positive (HHLA2 (+)/PD-L1 (−) or HHLA2 (−)/PD-L1 (+)); type BIII: TILs positive and both positive (HHLA2(+)/PD-L1(+)). ccRCC, clear cell renal cell carcinoma; HHLA2, human endogenous retrovirus-H long terminalrepeat-associating protein 2; OS, overall survival; PD-L1, programmed death 1 ligand 1; PFS, progression-free survival; TIL, tumor-infiltrating lymphocyte.

To explore the prognostic significance of HHLA2 and PD-L1, a univariate Cox proportional hazards regression was performed. HHLA2 (+) patients had significantly increased risks of disease progression and all causes of death compared with HHLA2 (−) patients (both in the training cohort (PFS: HR=3.23, 95% CI: 1.90 to 5.50, p<0.001; OS: HR=3.98, 95% CI: 2.15 to 7.37, p<0.001) and in the validation cohort (PFS: HR=2.17, 95% CI: 1.30 to 3.62, p=0.003, OS: HR=3.19, 95% CI: 1.82 to 5.58, p<0.001) (table 2). Kaplan-Meier analysis revealed that HHLA2 positivity was significantly associated with worse PFS and OS in the training cohort (training cohort, PFS: p<0.001; OS: p<0.001) and in the validation cohort (PFS: p<0.001, OS: p<0.001) (figure 2A).

**Table 2:** Univariate analyses of prognostic factors correlated with PFS and OS

Variables	Progression-free survival				Over survival			
	Training cohort		Validation cohort		Training cohort		Validation cohort	
	HR (95% CI)	P value	HR (95% CI)	P value	HR (95% CI)	P value	HR (95% CI)	P value
Age (y) ≥53/<53	1.251 (0.73 to 2.15)	0.415	1.55 (00.93 to 2.59)	0.091	1.57 (0.89 to 2.80)	0.122	1.58 (0.92 to 2.71)	0.096
Gender male/female	0.97 (0.56 to 1.70)	0.926	0.99 (0.57 to 1.75)	0.986	1.03 (0.58 to 1.82)	0.928	1.19 (90.67 to 2.11)	0.561
Necrosis present/absent	2.18 (1.28 to 3.74)	**0.004**	2.22 (1.30 to 3.77)	**0.003**	1.96 (1.12 to 3.43)	**0.018**	2.42 (1.39 to 4.20)	**0.002**
MVI present/absent	2.97 (1.62 to 5.46)	**<** **0.001**	3.46 (1.98 to 6.07)	**<** **0.001**	2.15 (1.10 to 4.20)	**0.024**	3.43 (1.90 to 6.18)	**<** **0.001**
Fuhrman grade high/low	3.97 (2.30 to 6.85)	**<** **0.001**	3.45 (2.06 to 5.77)	**<** **0.001**	3.74 (2.13 to 6.57)	**<** **0.001**	3.49 (2.03 to 6.02)	**<** **0.001**
TNM stage high/low	7.92 (4.46 to 14.08)	**<** **0.001**	3.77 (2.27 to 6.25)	**<** **0.001**	7.14 (3.98 to 12.83)	**<** **0.001**	3.93 (2.30 to 6.19)	**<** **0.001**
HHLA2 positive/negative	3.23 (1.90 to 5.50)	**<** **0.001**	2.17 (1.30 to 3.62)	**0.003**	3.98 (2.15 to 7.37)	**<** **0.001**	3.19 (1.82 to 5.58)	**<** **0.001**
PD-L1 positive/negative	3.26 (1.91 to 5.55)	**<** **0.001**	2.77 (1.66 to 4.61)	**<** **0.001**	3.02 (1.73 to 5.24)	**<** **0.001**	2.92 (1.71 to 4.99)	**<** **0.001**

*P values <0.05 in bold are statistically significant.

HHLA2, human endogenous retrovirus-H long terminal repeat-associating protein 2; MVI, microvascular invasion; OS, overall survival; PD-L1, programmed death 1 ligand 1; PFS, progression-free survival.

In line with HHLA2, PD-L1 (+) patients suffer much higher disease progression rates and mortality rates compared with PD-L1 (−) patients in both cohorts (PFS: HR=3.26, 95% CI: 1.91 to 5.55, p<0.001; OS: HR=3.02, 95% CI: 1.73 to 5.24, p<0.001 in the training cohort, PFS: HR=2.77, 95% CI: 1.66 to 4.61, p<0.001; OS: HR=2.92, 95% CI: 1.71 to 4.99, p<0.001 in the validation cohort) (table 2). Kaplan-Meier analysis also suggested that PD-L1 positivity was significantly associated with worse PFS and OS (figure 2B, training cohort, PFS: p<0.001, OS: p<0.001; validation cohort, PFS: p<0.001, OS: p<0.001).

Furthermore, multivariate analysis showed that both HHLA2 and PD-L1 were independent predictors for PFS and OS in the training cohort (HHLA2, PFS: HR=2.10, 95% CI: 1.11 to 3.99, p=0.023, OS: HR=2.14, 95% CI: 1.11 to 4.15, p=0.024; PD-L1, PFS: HR=2.28, 95% CI: 1.30 to 3.99, p=0.004, OS: HR=2.27, 95% CI: 1.26 to 4.09, p=0.006) (table 3). However, only HHLA2 was an independent predictor for PFS and OS in the validation cohort (HHLA2, PFS: HR=2.12, 95% CI: 1.22 to 3.71, p=0.008, OS: HR=2.08, 95% CI: 1.16 to 3.74, p=0.014) (table 3).

**Table 3:** Multivariate analyses of prognostic factors correlated with PFS and OS

Variables	Progression-free survival				Over survival			
	Training cohort		Validation cohort		Training cohort		Validation cohort	
	HR (95% CI)	P value	HR (95% CI)	P value	HR (95% CI)	P value	HR (95% CI)	P value
Necrosis present/absent	1.00 (0.55 to 1.80)	0.995	1.27 (0.71 to 2.27)	0.429	0.97 (0.53 to 1.77)	0.911	1.35 (0.73 to 2.51)	0.335
MVI present/absent	0.95 (0.48 to 1.88)	0.872	1.64 (0.90 to 2.99)	0.108	0.58 (0.27 to 1.25)	0.167	1.45 (0.77 to 2.76)	0.252
Fuhrman grade high/low	1.45 (0.77 to 2.73)	0.251	1.93 (1.09 to 3.39)	**0.023**	1.50 (0.79 to 2.87)	0.217	2.04 (1.13 to 3.68)	**0.018**
TNM stage high/low	5.14 (2.67 to 9.91)	**<** **0.001**	2.00 (1.14 to 3.51)	**0.016**	5.26 (2.72 to 10.18)	**<** **0.001**	2.02 (1.10 to 3.69)	**0.023**
HHLA2 positive/negative	2.10 (1.11 to 3.99)	**0.023**	2.12 (1.22 to 3.71)	**0.008**	2.14 (1.11 to 4.15)	**0.024**	2.08 (1.16 to 3.74)	**0.014**
PD-L1 positive/negative	2.28 (1.30 to 3.99)	**0.004**	1.61 (0.93 to 2.77)	0.087	2.27 (1.26 to 4.09)	**0.006**	1.66 (0.94 to 2.95)	0.081

*P values <0.05 in bold are statistically significant.

HHLA2, human endogenous retrovirus-H long terminal repeat-associating protein 2; MVI, microvascular invasion; OS, overall survival; PD-L1, programmed death 1 ligand 1; PFS, progression-free survival.

### Co-expression of HHLA2 and PD-L1 in ccRCC and prognostic significance

Considering that both HHLA2 and PD-L1 belong to the B7 family and both have an inhibitory function on CD4^+^ or CD8^+^ T cells, we attempted to explore the prognostic impact of HHLA2/PD-L1 co-expression in ccRCC. In both cohorts, 40 (19.4%) and 36 (18.3%) patients were HHLA2 (+)/PD-L1 (+), 51 (24.8%) and 45 (22.8%) patients were HHLA2 (+)/PD-L1 (−), 28 (13.6%) and 27 (13.7%) patients were HHLA2 (−)/PD-L1 (+), and 87 (42.2%) and 89 (45.2%) patients were HHLA2 (−)/PD-L1 (−), respectively (table 1). As Kaplan-Meier analysis demonstrated no significant difference for PFS and OS between patients with HHLA2 (+)/PD-L1 (−) and patients with HHLA2 (−) /PD-L1 (+) in both cohorts (online supplementary figure S1), patients were divided into three groups: group I, both negative (HHLA2 (−)/PD-L1 (−)); group II, single positive (HHLA2 (+)/PD-L1 (−) or HHLA2 (−)/PD-L1 (+)); and group III, both positive (HHLA2 (+)/PD-L1 (+)).

10.1136/jitc-2019-000157.supp2 Supplementary data

As illustrated in the online supplementary table S3, group III was closely associated with necrosis (p=0.001 or <0.001) and advanced TNM stage (both p<0.001) in the training and validation cohorts, and was additionally associated with MVI (p<0.001) and high Fuhrman grade (p<0.001) in the training cohort. Kaplan-Meier analysis demonstrated that both PFS and OS in group III were significantly reduced compared with group I and group II in both cohorts (figure 2C). Importantly, multivariate analysis revealed that the HHLA2 (+)/PD-L1 (+) still had significant impact on PFS and OS (PFS: HR=2.29, 95% CI: 1.53 to 3.41, p<0.001; OS: HR=2.31, 95% CI: 1.52 to 3.50, p<0.001 in the training cohort, PFS: HR=1.84, 95% CI: 1.28 to 2.65, p=0.001; OS: HR=1.86, 95% CI: 1.26 to 2.74, p=0.002 in the validation cohort) (table 4).

**Table 4:** Multivariate analyses of prognostic factors correlated with PFS and OS

Variables	Training cohort				Validation cohort			
	PFS		OS		PFS		OS	
	HR (95% CI)	P value	HR (95% CI)	P value	HR (95% CI)	P value	HR (95% CI)	P value
Necrosis present/absent	0.99 (0.55 to 1.79)	0.982	0.96 (0.53 to 1.76)	0.899	1.27 (0.71 to 2.28)	0.430	1.36 (0.74 to 2.53)	0.324
MVI present/absent	0.95 (0.48 to 1.87)	0.888	0.58 (0.28 to 1.23)	0.158	1.63 (0.89 to 2.98)	0.112	1.44 (0.76 to 2.72)	0.269
Fuhrman grade high/low	1.43 (0.77 to 2.66)	0.265	1.48 (0.78 to 2.81)	0.226	1.93 (1.09 to 3.40)	**0.024**	2.04 (1.12 to 3.69)	**0.019**
TNM stage high/low	5.06 (2.64 to 9.72)	**<** **0.001**	5.19 (2.69 to 10.01)	**<** **0.001**	1.98 (1.13 to 3.49)	**0.018**	2.00 (1.09 to 3.67)	**0.025**
HHLA2/PD-L1 group III/II/I	2.29 (1.53 to 3.41)	**<** **0.001**	2.31 (1.52 to 3.50	**<** **0.001**	1.84 (1.28 to 2.65)	**0.001**	1.86 (1.26 to 2.74)	**0.002**

*P values <0.05 in bold are statistically significant.

HHLA2, human endogenous retrovirus-H long terminal repeat-associating protein 2; MVI, microvascular invasion; OS, overall survival; PD-L1, programmed death 1 ligand 1; PFS, progression-free survival.

### Relationship between HHLA2/PD-L1 co-expression and the infiltration of CD4^+^/CD8^+^ TILs in ccRCC

**Figure 3:**
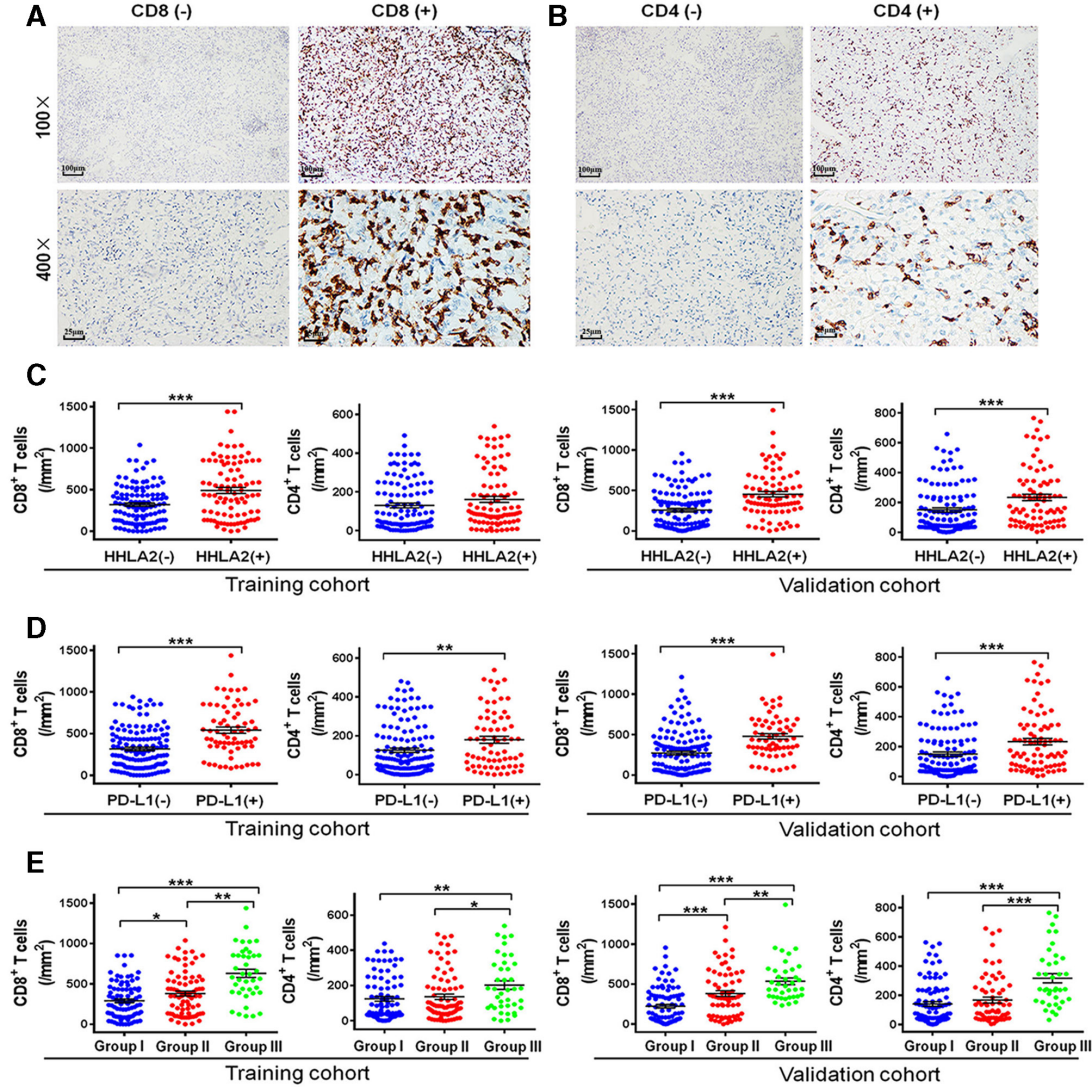
Representative micrographs of CD8 (A) and CD4 (B) expression and the corresponding negative controls within the tumor. Scatter plot depicted the correlation between HHLA2 and PD-L1 expression and classic subsets of T cells. (C) The correlation of HHLA2 expression and CD8^+^ T cells and CD4^+^ T cells in the training cohort and validation cohort. (D) The correlation of PD-L1 expression and CD8^+^ T cells and CD4^+^ T cells in the training cohort and validation cohort. (E) The correlation of the HHLA2/PD-L1 co-expression and CD8^+^ T cells and CD4^+^ T cells in the training cohort and validation cohort. Group I: HHLA2 (−)/PD-L1 (−); group II: HHLA2 (+)/PD-L1 (−) or HHLA2 (−)/PD-L1 (+); group III: HHLA2 (+)/PD-L1 (+). HHLA2, human endogenous retrovirus-H long terminalrepeat-associating protein 2; PD-L1, programmed death 1 ligand 1.

As HHLA2 and PD-L1 expressions are induced by immunoregulatory factors mainly produced by TILs, we performed IHC to detect the density of CD4^+^ and CD8^+^ TILs in ccRCC (figure 3A, B). As shown in figure 3C, the density of CD8^+^ TILs in the HHLA2 (+) tumors was significantly higher than that in HHLA2 (−) tumors in both cohorts (p<0.001). No significant association was found between CD4^+^ TILs and HHLA2 expression in the training cohort, whereas a positive association was detected between high HHLA2 expression and CD4^+^ TILs in the validation cohort (figure 3C). There were higher densities of CD8^+^ and CD4^+^ TILs in PD-L1 (+) tumors compared with the PD-L1 (−) tumors (figure 3D). Furthermore, the infiltration of CD8^+^ (both p<0.01) and CD4^+^ TILs (p<0.05 or<0.01) was strongest in group III (HHLA2 (+)/PD-L1 (+)) in both the training and validation cohorts (figure 3E).

### Immune classification for ccRCC

Considering the important predictive value of HHLA2/PD-L1 co-expression for both PFS and OS, and the significant correlation between HHLA2/PD-L1 co-expression and high density of TILs in ccRCC, we classified the patients into six types, based on TILs and HHLA2/PD-L1 co-expression, to provide rationale for immunotherapy. Of the patients in both cohorts, 19.9% (41/206) and 22.8% (45/197) belong to type AI, defined as negative TILs and both negative (HHLA2 (−)/PD-L1 (−)); 12.7% (26/206) and 14.2% (28/197) belong to type AII, defined as negative TILs and single positive (HHLA2 (+)/PD-L1 (−) or HHLA2 (−)/PD-L1 (+)); 1.9% (4/206) and 2.5% (5/197) belong to type AIII, defined as negative TILs and both positive(HHLA2 (−)/PD-L1 (−)); 21.8% (45/206) and 22.3% (44/197) belong to type BI, defined as positive TILs and both negative; 25.7% (53/206) and 22.3% (44/197) belong to type BII, defined as positive TILs and single positive; 18.0% (37/206) and 15.0% (31/197) belong to type BIII, defined as negative TILs and both positive (online supplementary table S4). As shown in figure 2D, in the TILs-positive subgroups, Kaplan-Meier analysis demonstrated that both PFS and OS in group BIII were significantly reduced compared with group BI and group BII in both cohorts. However, in the TILs-negative subgroup, no significant difference for either PFS or OS was detected among the three groups (online supplementary figure S2).

10.1136/jitc-2019-000157.supp3 Supplementary data

## Discussion

HHLA2, a newly identified B7 family member, is widely expressed with positive rates ranging from 0% to 70%,^16 27^ and associated with poor prognosis in various cancers, such as lung cancer, cholangiocarcinoma, pancreatic carcinoma, and osteosarcoma.^20 27–29^ Previous studies reported that high expression of HHLA2 was associated with poor prognosis in ccRCC; moreover, high HHLA2 expression was an independent risk factor for the prognosis of ccRCC.^21 22^ However, due to limited sample size,^21 22^ especially in cases at the advanced clinical stage,^21^ these two studies could not provide sufficient evidence to completely illustrate the role of HHLA2 in the prognosis of patients with ccRCC. Herein, based on two large cohorts with 403 cases in total, we analyzed the expression pattern of HHLA2 and its clinical significance in ccRCC, and found that the positive rate of HHLA2 expression in ccRCC was over 40% and that of HHLA2 (+) was associated with shorter PFS and OS, in line with previous literature. Furthermore, high expression of HHLA2 was an independent prognostic factor in both cohorts after adjusting for necrosis, MVI, Fuhrman grade, and TNM stage. Notably, the expression of HHLA2 was more frequent than PD-L1, another renowned inhibitory costimulatory factor, consistent with prior literature,^15 20^ which indicates that HHLA2 may be considered as a potential target for immunotherapy.

Although the expression of both PD-L1 and HHLA2 could be induced by immunoregulatory factors within the tumor microenvironment, the cells with HHLA2 (+)/PD-L1 (+) are low (16%) and quite part of the cases with PD-L1 negative contained a high expression of HHLA2 in lung cancer.^15^ Similarly, in our results, we found that the percentages of HHLA2 (+)/PD-L1 (+) were only 19.4% in the training cohort and 19.2% in the validation cohort, and >30% of PD-L1 (−) cases have high expression of HHLA2. Considering the different distributions between HHLA2 and PD-L1 expression, we were the first to investigate the clinical significance of HHLA2 and PD-L1 co-expression in ccRCC. We found that HHLA2/PD-L1 co-expression was significantly associated with the TNM stage, which acted as an independent prognostic factor for shorter PFS and OS. These results provide some useful guidelines for immunotherapy: patients with HHLA2 (+)/PD-L1 (+) may benefit from the dual blockade of PD-L1 and HHLA2; patients with one immune marker positive (PD-L1 or HHLA2) may need corresponding immune blockage to improve efficacy; for patients with no immune maker positive, the blockade of PD-L1 or HHLA2 may be inefficient and immunotherapy targeting another checkpoint, such as IDO1, TIM3, B7-H3, may be needed.

Till now, the relationship between HHLA2 expression and immune cell infiltration in various cancers is still debatable. Cheng *et al* demonstrated that HHLA2 high expression was significantly associated with high TIL density in lung cancer,^15^ however, Koirala *et al* reported that no significant correlation was detected between HHLA2 expression and TILs in osteosarcoma.^27^ Furthermore, a negative association was observed between HHLA2 overexpression and cytotoxic T cells.^20^ In renal cancer, Chen *et al* documented that there was no significant association between HHLA2 overexpression and the infiltrating intensity of CD8^+^ T cells, based on a small cohort of study.^21^ In the present study, we found that HHLA2 overexpression was significantly associated with a high density of CD8^+^ TILs, but not with the infiltration of CD4^+^ TILs. These data suggested that HHLA2 can be induced by interferon-gamma (IFN-γ) which mainly secreted by CD8^+^ TILs. Nevertheless, similar to PD-L1, HHLA2 could decrease both the CD8^+^ and CD4^+^ TILs proliferation and functionally inhibit the production of several cytokines, such as IFN-γ, tumor necrosis factor-alpha, interleukin (IL)-5, IL-10, IL-13, and IL-17A. Therefore, through HHLA2 blockage, HHLA2(+) patients could benefit from immunotherapy.

According to Teng *et al*, a tumor is classified into four groups based on TIL density and PD-L1 expression, including: adaptive immune resistance, immunological ignorance, tolerance, and intrinsic induction.^8^ However, the feasibility of Teng’s theory in ccRCC has not been confirmed. The expression of PD-L1 was not the most prevalent among B7 family members. For example, in PD-L1-negative lung cancer and cholangiocarcinoma, HHLA2 was more prevalent than PD-L1; and HHLA2 overexpression was common.^15 20^ Therefore, this theory may overestimate the proportions of immunological ignorance and tolerance patterns, and inversely underestimate the proportion of adaptive immune resistance and intrinsic induction patterns. Hence, it may be much more rational to classify by TILs via the combination PD-L1 and HHLA2 or in combination with other B7 family members.

In this study, we classified patients with ccRCC into six groups based on HHLA2/PD-L1 co-expression and TILs. In this new classification, our results demonstrated that in TIL-positive groups, HHLA2/PD-L1 co-expression displayed significant prognostic value for PFS and OS; however, no significant difference for PFS and OS was detected in TIL-negative groups. These results suggested that both HHLA2 and PD-L1 perform immunosuppressive functions that mainly depend on suppression of the TILs function.

There are several limitations involved in this study. First, although we conducted a dual-center study based on a large number of cases, this was a retrospective study and the selection bias was inevitable. Second, we only combined HHLA2 and PD-L1 to conduct immune classification. Actually, other B7 family members, such as B7-H3 and B7x, or other immunosuppressive factors such as metabolites (eg, adenosine, IDO), also contribute to immunosuppression in the tumor microenvironment.^30–32^ Therefore, an immunophenotyping system containing other B7 family members is needed to guide clinical immunotherapy. Finally, because fewer patients were TIL negative, especially patients in type AIII subgroup, confirmation from a larger cohort is needed to determine whether HHLA2/PD-L1 co-expression displays no significant prognostic value for PFS and OS in TIL-negative groups.

## Conclusion

Herein, we elucidated that HHLA2 expression was more prevalent than PD-L1 in ccRCC. HHLA2/PD-L1 co-expression was an independent predictor of poor prognosis, and these results may provide insight into effective strategies for combination therapy in ccRCC.
