# Correction: *HHLA2 and PD-L1 co-expression predicts poor prognosis in patients with clear cell renal cell carcinoma*

**DOI:** 10.1136/jitc-2019-000157corr1

**Published:** 2020-04-28

Zhou Q, Li K, Chen X, et al. HHLA2 and PD-L1 co-expression predicts poor prognosis in patients with clear cell renal cell carcinoma. J ImmunoTherap Cancer 2020;8:e000157. doi: 10.1136/jitc-2019-000157

In this article, the last panels in figure 3C and 3D incorrectly show the same image. The corrected figure 3 is shown below


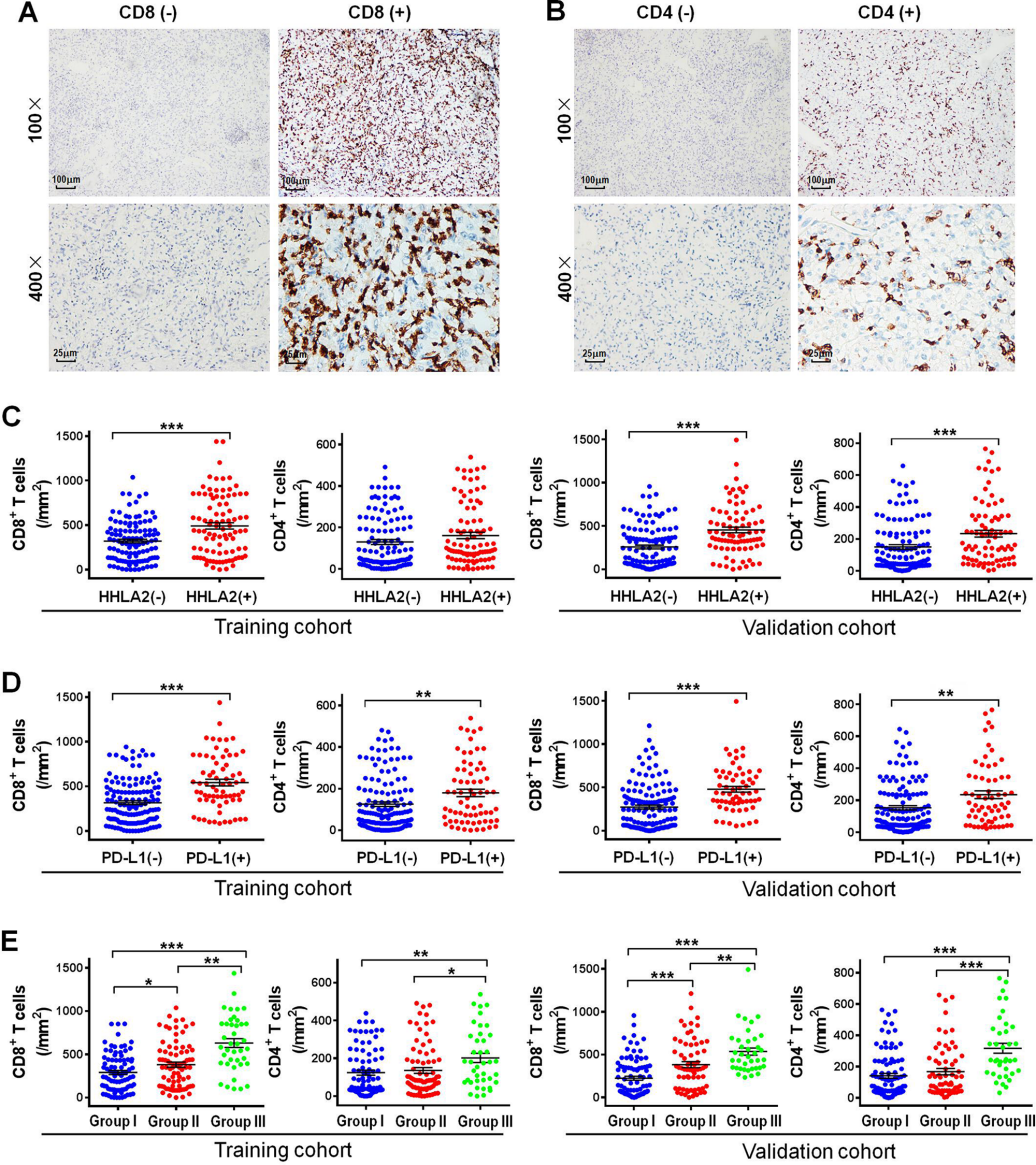
Figure 3. Representative micrographs of CD8 (A) and CD4 (B) expression and the corresponding negative controls within the tumor. Scatter plot depicted the correlation between HHLA2 and PD-L1 expression and classic subsets of T cells. (C) The correlation of HHLA2 expression and CD8+ T cells and CD4+ T cells in the training cohort and validation cohort. (D) The correlation of PD-L1 expression and CD8+ T cells and CD4+ T cells in the training cohort and validation cohort. (E) The correlation of the HHLA2/PD-L1 co-expression and CD8+ T cells and CD4+ T cells in the training cohort and validation cohort. Group I: HHLA2 (−)/PD-L1 (−); group II: HHLA2 (+)/PD-L1 (−) or HHLA2 (−)/PD-L1 (+); group III: HHLA2 (+)/PD-L1 (+). HHLA2, human endogenous retrovirus-H long terminal repeat-associating protein 2; PD-L1, programmed death 1 ligand 1.

Additionally, in the Results section titled ‘Immune classification for ccRCC’ the sentence “1.9% (4/206) and 2.5% (5/197) belong to type AIII, defined as negative TILs and both positive(HHLA2 (−)/PD-L1 (−)); 21.8% (45/206) and 22.3% (44/197) belong to type BI, defined as posi- tive TILs and both negative; 25.7% (53/206) and 22.3% (44/197) belong to type BII, defined as positive TILs and single positive; 18.0% (37/206) and 15.0% (31/197) belong to type BIII, defined as negative TILs and both positive (online supplementary table 4)” should read “1.9% (4/206) and 2.5% (5/197) belong to type AIII, defined as negative TILs and both positive(HHLA2 (+)/PD-L1 (+)); 21.8% (45/206) and 22.3% (44/197) belong to type BI, defined as posi- tive TILs and both negative; 25.7% (53/206) and 22.3% (44/197) belong to type BII, defined as positive TILs and single positive; 18.0% (37/206) and 15.0% (31/197) belong to type BIII, defined as positive TILs and both positive (online supplementary table 4).”

