# Supplementary material for: HHLA2 and PD-L1 co-expression predicts poor prognosis in patients with clear cell renal cell carcinoma
Source: J Immunother Cancer. 2020 Jan 19;8(1):e000157. doi: 10.1136/jitc-2019-000157 (PMC7057441; doi:10.1136/jitc-2019-000157)
Supplement: Supplementary data [file jitc-2019-000157supp001.pdf]

Table S1. Basic characteristics of patients

Variables		Training cohort (n=206)	Validation cohort (n=197)
		Number of cases (%)	Number of cases (%)
Age	54(19-84)		
(y, Median	<53	90(43.7%)	102(51.8%)
range)	≥53	116(56.3%)	95(48.2%)
Gender	Male	134(65.0%)	141(71.6%)
	Female	72(35.0%)	56(28.4%)
T stage	T1	116(56.3%)	118(59.9%)
	T2	48(23.3%)	39(19.8%)
	T3	32(15.5%)	33(16.8%)
	T4	10(4.9%)	7(3.6%)
N stage	N0	187(90.8%)	181(91.9%)
	N1	19(9.2%)	16(8.1%)
M stage	M0	181(87.9%)	185(93.9%)
	M1	25(12.1%)	12(6.1%)
Necrosis	Absent	149(72.3%)	151(76.6%)
	Present	57(27.7%)	46(23.4%)
MVI	Absent	179(86.9%)	169(85.8%)
	Present	27(13.1%)	28(14.2%)
Fuhrman grade	G1	42(20.4%)	44(22.3%)
	G2	94(45.6%)	85(43.1%)
	G3	55(26.7%)	49(24.9%)
	G4	15(7.3%)	19(9.6%)
HHLA2	Negative	115(55.8%)	116(58.9%)
	Positive	91(44.2%)	81(41.1%)
PD-L1	Negative	138(67.0%)	134(68.0%)
	Positive	68(33.0%)	63(32.0%)

Abbreviations: MVI, microvascular invasion, HHLA2: Human endogenous retrovirus-H long terminal repeat-associating protein 2, PD-L1: Programmed death 1 ligand 1

Table S2. The association of PD-L1 expression level with clinicopathological characteristics

Variables	Training cohort(n=206)				Validation cohort(n=197)			
	Patients	PD-L1 expression			Patients	PD-L1 expression		
	NO.	Negative	Positive	*p*-value	NO.	Negative	Positive	*p*-value
Age(y)				0.268				0.301
<53	90	64	26		102	66	36	
≥53	116	74	42		95	68	27	
Gender				0.702				0.479
Male	134	91	43		141	98	43	
Female	72	47	25		56	36	20	
Necrosis				**0.041**				**0.001**
Absent	149	106	43		151	112	39	
Present	57	32	25		46	22	24	
MVI				**<0.001**				0.077
Absent	179	128	51		169	119	50	
Present	27	10	17		28	15	13	
Fuhrman grade				**0.002**				**0.003**
Low (1,2)	136	101	35		129	97	32	
High (3,4)	70	37	33		68	37	31	
TNM stage				**0.003**				**0.002**
Low (I, II)	146	107	39		146	108	38	
High (III, IV)	60	31	29		51	26	25	

Abbreviations: MVI microvascular invasion; p-value$<$*0.05 marked in bold font shows statistical significance.*

Table S3. The association of HHLA2/PD-L1 co-expression level with clinicopathological characteristics in training cohort and validation cohort.

Variables	Training cohort					Validation cohort				
	Patients NO.	HHLA2/PD-L1 co-expression				Patients NO.	HHLA2/PD-L1 co-expression			
		Group I	Group II	Group III	*p*-value		Group I	Group II	Group III	*p*-value
Age(y)					0.229					0.885
<53	90	44	30	16		102	47	34	21	
≥53	116	43	29	24		95	46	32	17	
Gender					0.698					0.051
Male	134	54	54	26		141	74	41	26	
Female	72	33	25	14		56	19	25	12	
Necrosis					**0.001**					**<0.001**
Absent	149	75	51	23		151	83	46	22	
Present	57	12	28	17		46	10	20	16	
MVI					**<0.001**					0.385
Absent	179	82	70	27		169	82	57	30	
Present	27	5	9	13		28	11	9	8	
Fuhrman grade					**<0.001**					0.310
Low (1,2)	136	75	50	19		129	66	40	23	
High (3,4)	70	12	35	23		68	27	26	15	
TNM stage					**<0.001**					**0.011**
Low (I, II)	146	77	77	59		146	78	42	26	
High (III, IV)	60	10	29	21		51	15	24	12	

Table S4. Distribution of types of microenvironment in renal cell carcinoma based on HHLA2/PD-L1co-expression of tumor cells and density of CD4 plus CD8-positive TILs

Intra-tumoral TILs	Training cohort			Validation cohort		
	HHLA2/PD-L1 co-expression			HHLA2/PD-L1 co-expression		
	Group I (%)	Group II (%)	Group III (%)	Group I (%)	Group II (%)	Group III (%)
Negative	41(19.9)	26(12.7)	4(1.9)	45(22.8)	28(14.2)	5(2.5)
Positive	45(21.8)	53(25.7)	37(18.0)	44(22.4)	44(22.4)	31(15.7)

Group I, both negative (HHLA2(-)/PD-L1(-)); Group II, single positive (HHLA2 (+)/PD-L1(-) or HHLA2(-)/PD-L1(+)); and Group III, both positive (HHLA2 (+)/PD-L1(+)).

Figure Legends

Figure S1. Kaplan Meier survival curves for PFS and OS of patients with ccRCC according to HHLA2/PD-L1 co-expression in training cohort(a) and validation cohort(b).

Figure S2. Kaplan Meier survival curves for PFS and OS of patients with ccRCC according to HHLA2/PD-L1 co-expression in training cohort(a) and validation cohort(b).
