# Supplementary figures and images for: HHLA2 and PD-L1 co-expression predicts poor prognosis in patients with clear cell renal cell carcinoma
Source: J Immunother Cancer. 2020 Jan 19;8(1):e000157. doi: 10.1136/jitc-2019-000157 (PMC7057441; doi:10.1136/jitc-2019-000157)

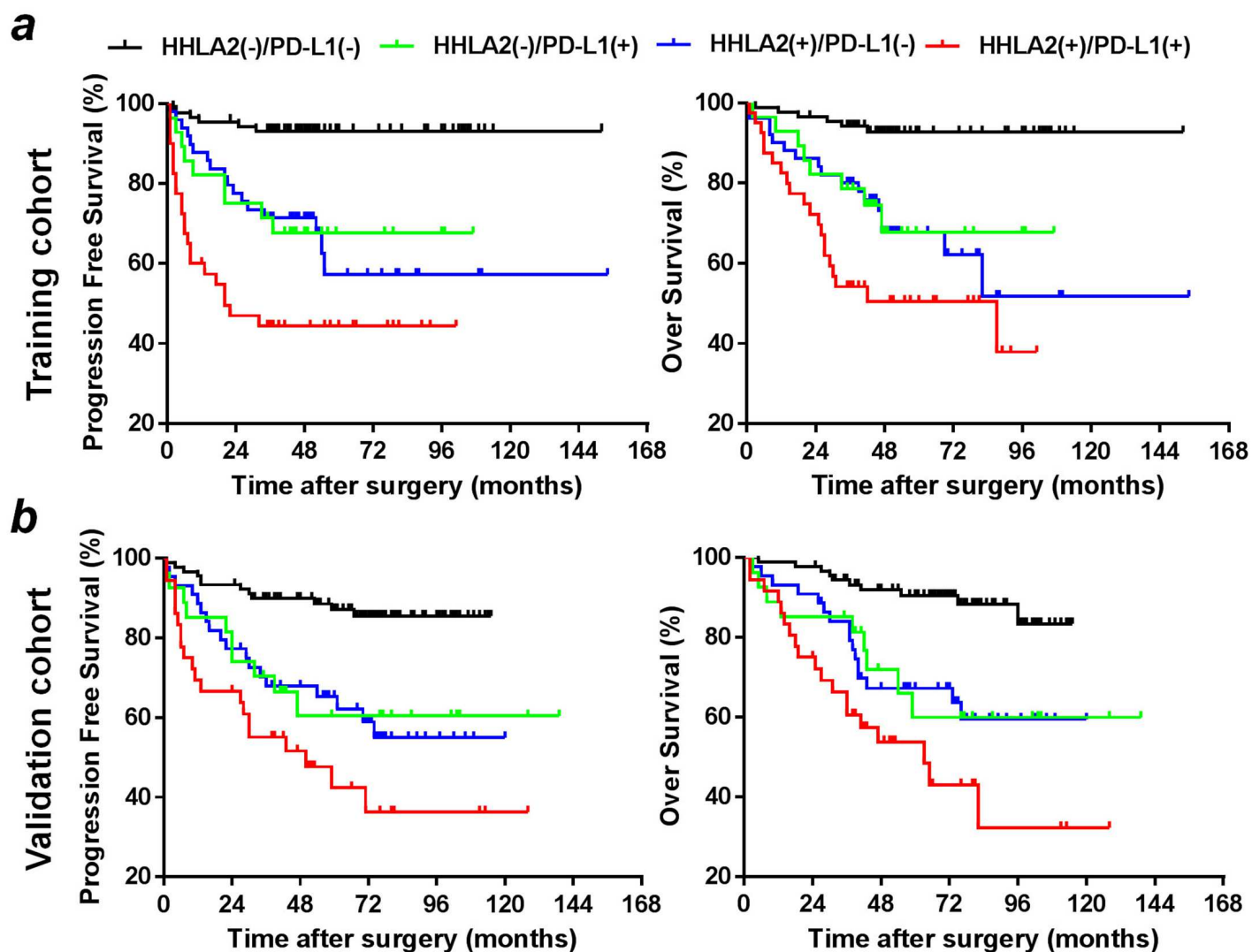

Supplement: Supplementary data [file jitc-2019-000157supp002.pdf]

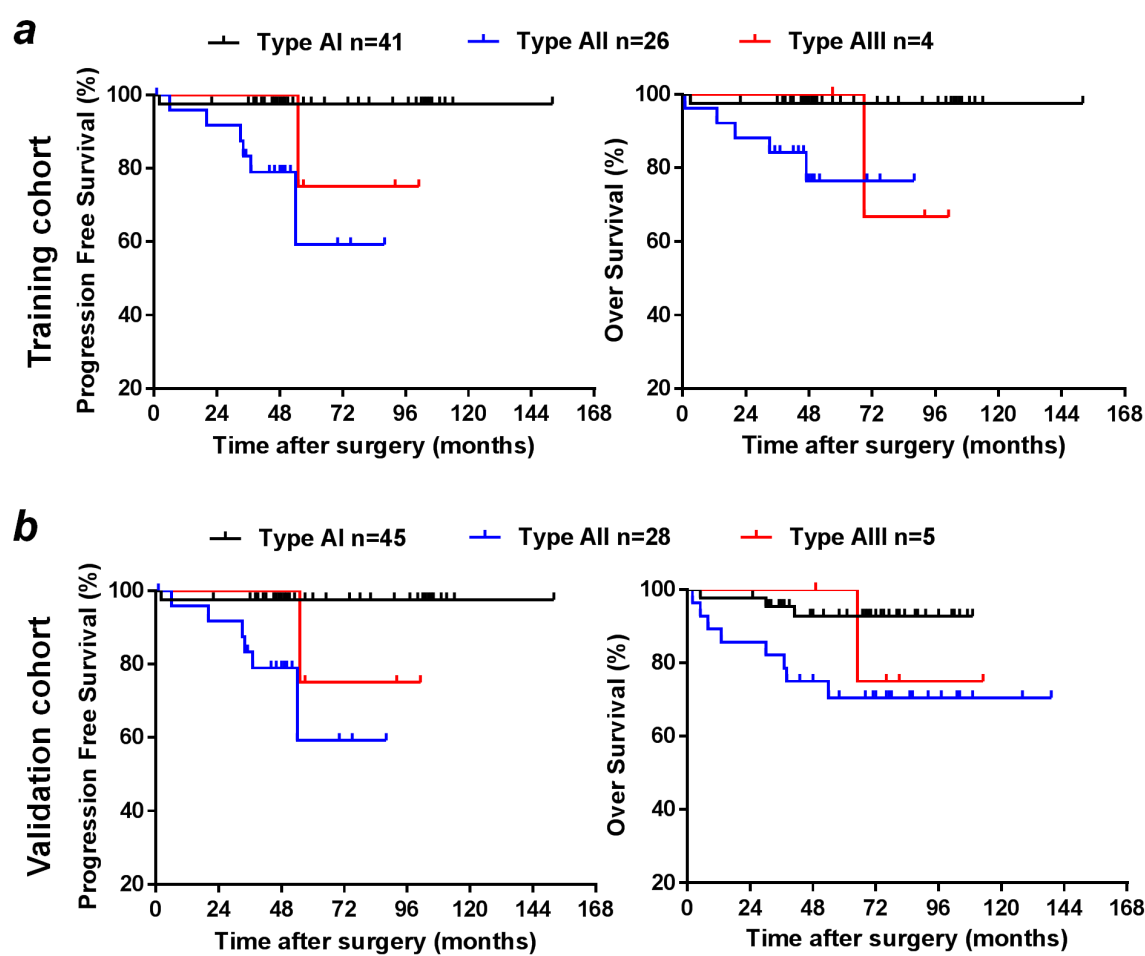

Supplement: Supplementary data [file jitc-2019-000157supp003.pdf]
